# Amplified Loci on Chromosomes 8 and 17 Predict Early Relapse in ER-Positive Breast Cancers

**DOI:** 10.1371/journal.pone.0038575

**Published:** 2012-06-13

**Authors:** Erhan Bilal, Kristen Vassallo, Deborah Toppmeyer, Nicola Barnard, Inga H. Rye, Vanessa Almendro, Hege Russnes, Anne-Lise Børresen-Dale, Arnold J. Levine, Gyan Bhanot, Shridar Ganesan

**Affiliations:** 1 Rutgers, The State University of New Jersey, Piscataway, New Jersey, United States of America; 2 Cancer Institute of New Jersey, New Brunswick, New Jersey, United States of America; 3 Robert Wood Johnson University Hospital, New Brunswick, New Jersey, United States of America; 4 Institute for Cancer Research, The Norwegian Radium Hospital, Oslo, Norway; 5 Dana Farber Cancer Institute, Harvard University, Boston, Massachusetts, United States of America; 6 Institute of Clinical Medicine, University of Oslo, Oslo, Norway; 7 Department of Medical Oncology, Hospital Clinic, Barcelona, Spain; 8 Institute for Advanced Study, Princeton, New Jersey, United States of America; H.Lee Moffitt Cancer Center & Research Institute, United States of America

## Abstract

Adjuvant hormonal therapy is administered to all early stage ER+ breast cancers, and has led to significantly improved survival. Unfortunately, a subset of ER+ breast cancers suffer early relapse despite hormonal therapy. To identify molecular markers associated with early relapse in ER+ breast cancer, an outlier analysis method was applied to a published gene expression dataset of 268 ER+ early-stage breast cancers treated with tamoxifen alone. Increased expression of sets of genes that clustered in chromosomal locations consistent with the presence of amplicons at 8q24.3, 8p11.2, 17q12 (HER2 locus) and 17q21.33-q25.1 were each found to be independent markers for early disease recurrence. Distant metastasis free survival (DMFS) after 10 years for cases with any amplicon (DMFS  = 56.1%, 95% CI  = 48.3–63.9%) was significantly lower (P  = 0.0016) than cases without any of the amplicons (DMFS  = 87%, 95% CI  = 76.3% –97.7%). The association between presence of chromosomal amplifications in these regions and poor outcome in ER+ breast cancers was independent of histologic grade and was confirmed in independent clinical datasets. A separate validation using a FISH-based assay to detect the amplicons at 8q24.3, 8p11.2, and 17q21.33-q25.1 in a set of 36 early stage ER+/HER2- breast cancers treated with tamoxifen suggests that the presence of these amplicons are indeed predictive of early recurrence. We conclude that these amplicons may serve as prognostic markers of early relapse in ER+ breast cancer, and may identify novel therapeutic targets for poor prognosis ER+ breast cancers.

## Introduction

Hormone therapy is widely used for treatment of estrogen receptor positive (ER+) breast cancer and has been shown to result in significantly improved survival and lower rates of recurrence (reviewed in [1], [2]). However, a significant subset of ER+ breast cancer patients treated with adjuvant hormone therapy suffer early disease recurrence. These poor prognosis ER+ tumors tend to have higher grade and show higher proliferative indices and may not be “addicted” to ER –dependent signalling, making them resistant to hormone therapy and prone to early relapse (reviewed in [3]–[6]). A better understanding of the mechanisms underlying the early relapse of some ER+ breast cancers may lead to better prognostic assays, and to new targeted therapeutic strategies for these poor prognosis cancers.

Several assays have been developed to distinguish ER+ patients likely to do well with hormonal therapy from those likely to have early disease progression. The best validated of these is the Oncotype Dx® assay [7] from Genomic Health, Inc., based on RT-PCR measurement of mRNA levels of 21 genes. ER+ breast cancer patients whose tumors have low ODx Recurrence Scores (RS) do well with adjuvant hormonal therapy alone, while tumors with high ODx RS are more likely to benefit from the addition of chemotherapy to hormonal therapy. Other panels of genes, such as the Genomic Grade Index panel [8], and clinical markers such as histological grade, are also used to classify patients into good or poor prognosis classes. In addition, molecular signatures from clustering methods applied to gene-expression data are also able to separate ER+ breast cancers into good prognosis (Luminal A) and poor prognosis (Luminal B) classes [9]–[11]. However, several studies have shown that, when the prognostic assays are compared to the gene expression based sub-classification of breast cancers, these assays are essentially identifying Luminal A tumors (low grade, highly ER+ breast cancers, HER2-) as being good prognosis, and Luminal B, ER+ breast cancers (which are ER+, mostly intermediate-to-high grade, some with HER2 amplification) as poor prognosis [12]–[14].

Although gene expression based assays such as Oncotype Dx have prognostic and predictive utility, they do not identify the biologic pathways driving resistance in the poor prognosis tumors. Moreover the optimal strategy for “Intermediate Risk” ODx RS, found in up to 30% of ER+ cancers, is not clear at present. In contrast, the presence of the HER2 amplicon, in ER+ breast cancers, has both clear prognostic value and identifies a clear and effective therapeutic target. ER+ breast cancers with HER2-amplification tend to have early recurrence if treated with hormonal therapy alone, likely because the activation of the HER2 pathway leads to independence from ER- mediated signalling (see reviews above, also [15], [16]). Moreover, therapy that specifically targets HER2 has been shown to dramatically improve outcome in HER2+ patients. Thus all breast cancers are now routinely tested for the presence of HER2 amplification.

As HER2 amplicon genes are part of the 21 gene panel used in determining the Oncotype Dx recurrence score (RS), breast cancers with HER2 amplification generally have high RS, high histological grade, and a high genomic grade and are easily and correctly identified as poor prognosis by the assay. However, the majority of poor prognosis ER+ cancers with high ODx RS do not have HER2 amplification [14]. Indeed only patients with ER+ tumors and no evidence of HER2 amplification have Oncotype DX assays performed in most clinical settings. At present there is little insight into the mechanism driving estrogen independence and growth in poor prognosis ER+/HER2- breast cancers.

In order to gain insight into the biology of these poor prognosis ER+/HER2- breast cancers, we analyzed a public gene expression data set of early stage ER+ breast cancers treated with tamoxifen using a novel method. Sets of outlier genes whose expression correlated with clinical outcome were analyzed to identify either molecular pathways or enrichment of chromosomal regions. Four separate regions of the genome were identified whose amplification was highly predictive of poor prognosis in early stage ER+ breast cancers treated with tamoxifen. As expected, one of these was the HER2 amplicon on 17q12 [17], [18]; validating our methods as being able to identify relevant amplicons. The other three amplification regions were in 8q24.3, 8p11.2 and 17q21.33-q25.1. Although these loci have previously been identified as regions of amplification in subsets of breast cancer [19], their association with tamoxifen resistance in ER+/HER2- breast cancers is novel. The presence of these amplicons in ER+/HER2- breast cancer and their association with poor prognosis was validated in several independent data sets [20]. Taken together, these findings demonstrate that these amplicons are strong predictors of early relapse in ER+ breast cancers.

## Results

### Outlier Genes and Patterns Associated with Tamoxifen Treatment Response

A gene expression dataset (published by Loi et al. [12], [21]) containing 268 patients with early stage ER+ breast cancers treated with local therapy and adjuvant tamoxifen with 9+ years of available clinical follow-up data, was analyzed. Clinical characteristics of this set have been previously described (Table S1).

Genes whose expression values were outliers in at least 10 samples in this dataset were identified and analyzed for their correlation with distant metastasis free survival. Outlier genes for which there was a significant difference in distant metastasis free survival between samples having outlier expression when compared to samples with normal expression, were identified and retained (see Methods for details). Table S2 has the set of outlier genes, hazard ratios, log-rank P values and outlier scores.

**Figure 1:**
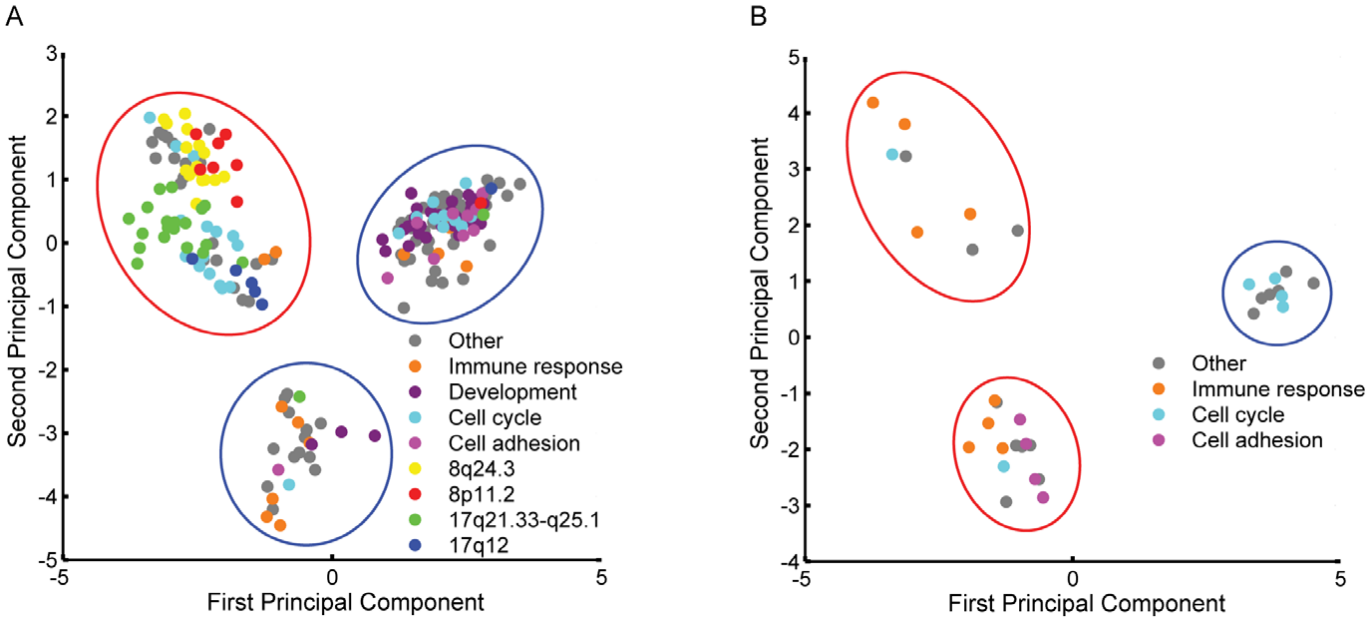
PCA plots of high and low outliers. Principal component analysis of high outlier genes (A) and low outlier genes (B) associated with differential distant metastasis free survival are shown. The figure represents the projection of each gene’s outlier profile on the first two principal components of the corresponding matrix. Gene clusters associated with good prognosis are circled in blue while gene clusters associated with bad prognosis are circled in red. Over-expressed genes associated with poor prognosis, which map to the chromosomal regions 8q24.3, 8p11.2, 17q21.33-q25.1 and 17q12, and are associated with specific GO pathways are labeled with different colors.

Principle component analysis (PCA) demonstrated that the outlier genes separated into 3 clusters (Figure 1A). Survival analysis of these clusters showed that one cluster contained genes whose over-expression associated with poor prognosis, and the other two contained genes over-expressed in good prognosis samples. The set of outlier genes in each cluster was analysed using Gene Ontology (GO) [22] to identify pathways and potential chromosomal amplifications associated with outcome (Table S3). Pathways enriched in over-expressed outliers associated with good prognosis included development, cell adhesion, and immune response genes. Of note, no clusters of outliers associated with good prognosis suggestive of an underlying amplicon were detected. Outlier genes whose over-expression was associated with poor prognosis had a significant enrichment of genes in cell cycle pathways. Analysis of outliers for clustering by chromosomal location identified putative amplification of four chromosomal regions associated with poor prognosis: 17q12, 17q21.33-q25.1, 8p11.2 and 8q24.3. The presence of genomic amplification in any of these regions leads to outlier expression of their genes, and is a marker of poor prognosis in ER+ breast cancer.

Cell cycle pathway outliers contained genes associated with proliferation and included many of the genes used to define the Genomic Grade Index [8]. This confirms prior observations that proliferation-associated genes are strong markers of poor prognosis in ER+ breast cancer. The known amplicon on 17q12 [23] associated with poor prognosis was also identified. This amplicon contains the HER2 gene and is known to be associated with relative resistance to hormonal therapy and poor prognosis in ER+ breast cancer. The other putative amplicons in 17q21.33-q25.1 [24]–[26], 8p11.2 [27], [28] and 8q24.3 [19] have been previously reported as amplified in subsets of breast cancers but their association with tamoxifen resistance in ER+ breast cancer is a novel finding. The full list of outlier genes identified in these amplified chromosomal regions is listed in Table 1, with potential oncogenes highlighted in red. Some of these genes have been previously identified as playing a role in tumorigenesis or cancer progression including, WHSC1L1 [29], [30], CLTC [31]–[33], HSF1 [34], and LSM1 [35]. Of note the FGFR1 which has been implicated in hormonal resistance in ER+ breast cancer [36], is present at the edges of the 8p11.2 amplicon, but is not present in our minimal amplicon defined by our analysis (see Table S2). Similarly MYC, another oncogene reported to induce hormone resistance in breast cancer, is upstream of the 8p24.3 amplicon defined by our analysis, and is not associated with poor outcome in this dataset (see Table S2).

**Table 1:** Over-expressed genes in chromosomal regions 17q12, 17q21.33-q25.1, 8p11.2 and 8q24.3 associated with early relapse in ER+ breast cancers treated with tamoxifen.

Gene	Hazard ratio	P value	Cytoband	Start	End
STARD3	2.23	4.19E−03	chr17q12	35,046,940	35,073,248
**ERBB2**	1.91	2.16E−02	chr17q12	35,110,005	35,122,109
GRB7	1.83	5.12E−02	chr17q12	35,152,029	35,156,782
GSDML	2.51	1.72E−03	chr17q12	35,326,079	35,328,194
PSMD3	1.78	3.57E−02	chr17q12	35,390,607	35,407,732
**PHB**	2.48	7.96E−04	chr17q21.33	44,836,413	44,847,246
SLC35B1	1.81	3.87E−02	chr17q21.33	45,133,688	45,140,281
SUPT4H1	2.20	5.11E−03	chr17q22	53,778,283	53,784,556
RAD51C	1.83	3.53E−02	chr17q22	54,124,987	54,127,694
**CLTC**	2.04	8.80E−03	chr17q23.1	55,052,102	55,126,906
PTRH2	1.96	1.27E−02	chr17q23.1	55,129,449	55,139,638
ABC1	2.15	1.36E−02	chr17q23.1	55,475,337	55,499,876
APPBP2	1.84	3.40E−02	chr17q23.2	55,875,300	55,958,365
TRIM37	1.84	3.39E−02	chr17q23.2	57,059,999	57,184,266
USP32	2.17	7.02E−03	chr17q23.2	58,254,691	58,469,586
CYB561	2.22	5.45E−03	chr17q23.3	58,864,245	58,869,052
CCDC44	1.89	3.97E−02	chr17q23.3	59,038,377	59,039,456
PSMC5	2.19	5.37E−03	chr17q23.3	59,258,832	59,263,111
PSMD12	1.98	1.94E−02	chr17q24.2	62,764,494	62,793,171
KPNA2	1.76	4.87E−02	chr17q24.2	66,031,848	66,042,970
ICT1	2.33	3.23E−03	chr17q25.1	70,520,374	70,528,950
ATP5H	1.91	3.55E−02	chr17q25.1	70,546,552	70,548,888
MRPS7	1.82	3.31E−02	chr17q25.1	70,769,394	70,773,734
SAP30BP	1.81	4.18E−02	chr17q25.1	71,175,038	71,214,431
SPFH2	1.85	2.55E−02	chr8p11.2	37,713,267	37,734,476
PROSC	2.19	6.19E−03	chr8p11.2	37,739,282	37,756,441
ASH2L	2.12	7.31E−03	chr8p11.2	38,082,214	38,116,216
**LSM1**	2.39	1.08E−03	chr8p11.2	38,140,017	38,153,183
**WHSC1L1**	2.28	3.90E−03	chr8p11.2	38,293,091	38,358,947
**BRF2**	3.04	2.62E−05	chr8p12	37,821,053	37,826,512
DDHD2	2.15	6.66E−03	chr8p12	38,208,356	38,239,442
UBE2V2	2.11	9.08E−03	chr8q11.21	49,083,545	49,136,681
ATP6V1H	2.45	1.47E−03	chr8q11.23	54,828,192	54,832,484
MRPL15	2.27	3.53E−03	chr8q11.23	55,210,341	55,223,011
COPS5	1.82	2.45E−02	chr8q13.2	68,117,869	68,136,905
TCEB1	2.74	3.53E−04	chr8q21.11	75,020,403	75,047,049
FAM82B	2.01	1.46E−02	chr8q21.3	87,555,453	87,590,037
UQCRB	2.13	8.20E−03	chr8q22	97,312,308	97,316,963
POLR2K	1.74	5.18E−02	chr8q22.2	101,232,001	101,235,407
ATP6V1C1	1.81	4.87E−02	chr8q22.3	104,102,463	104,152,473
EBAG9	1.80	4.48E−02	chr8q23	110,621,485	110,646,565
**YWHAZ**	2.85	1.30E−03	chr8q23.1	102,001,097	102,033,426
ENY2	2.70	3.08E−04	chr8q23.1	110,415,745	110,425,074
RAD21	1.95	2.60E−02	chr8q24	117,927,353	117,956,221
SQLE	2.13	1.49E−02	chr8q24.1	126,100,439	126,102,952
MRPL13	2.93	1.90E−04	chr8q24.12	121,477,267	121,526,557
SCRIB	2.39	5.07E−03	chr8q24.3	144,945,082	144,968,239
SIAHBP1	1.97	1.59E−02	chr8q24.3	144,970,536	144,983,471
GRINA	2.07	1.12E−02	chr8q24.3	145,136,247	145,139,570
EXOSC4	2.14	7.84E−03	chr8q24.3	145,205,516	145,207,538
CYC1	2.45	4.97E−03	chr8q24.3	145,221,982	145,224,415
SHARPIN	2.12	1.84E−02	chr8q24.3	145,225,527	145,230,852
C8orf30A	1.80	4.97E−02	chr8q24.3	145,264,659	145,267,608
BOP1	2.30	5.71E−03	chr8q24.3	145,456,867	145,485,928
**HSF1**	2.36	1.24E−02	chr8q24.3	145,497,218	145,498,193
FBXL6	2.62	6.13E−04	chr8q24.3	145,549,899	145,552,940
GPR172A	2.99	2.81E−04	chr8q24.3	145,553,131	145,555,738
VPS28	2.34	2.53E−03	chr8q24.3	145,619,807	145,623,174
RPL8	2.18	6.48E−03	chr8q24.3	145,985,957	145,988,332
ZNF7	2.17	8.43E−03	chr8q24.3	146,023,747	146,043,697
ZNF250	1.83	3.51E−02	chr8q24.3	146,076,967	146,079,026
C8orf33	1.94	1.63E−02	chr8q24.3	146,248,629	146,251,814

List of genes associated with early relapse on chromosomes 8 and 17. Highlighted in bold are cancer related genes of interest.

For under-expressed outliers, a similar analysis showed that relative under-expression of the cell cycle pathway was associated with good prognosis, while under-expression of the immune response and cell adhesion pathway was associated with poor prognosis (Figure 1B). This mirrors the results for over-expressed outlier genes and confirms the strong association of the cell cycle, immune response and cell adhesion pathways with prognosis in ER+ breast cancers.

### Correlations between Cell Cycle Pathway and Putative Amplicons in 17q12, 17q21.33-q25, 8p11.2, and 8q24.3

To examine the inter-relationship between the cell cycle pathway and the four potential amplicons identified by our analysis, a correlation matrix of all genes associated with poor outcome was computed (Figure S1). Correlations between the presence of each amplicon and any amplicon or the cell cycle pathway are shown in Table S4. We find that the cell cycle pathway correlates partly with all the amplicons (Figure S1, Table S4), suggesting that activation of cell cycle pathway is associated with chromosomal amplifications in 17q12, 17q21.33-q25, 8p11.2, and 8q24.3. Of note, the cell cycle genes themselves are not located in these amplicon regions.

The association between cell cycle and putative amplicons was further examined. Samples with enrichment of any of the four amplicons or the cell cycle pathway were identified by requiring at least 50% of gene markers in each group to be over-expressed, i.e. marked as a high outlier in the respective sample. It was found that in most samples (90.5%), over-expression of cell cycle genes display at least one of the four chromosomal amplifications, suggesting a causal relationship between tumor proliferation and the presence of these amplicons.

However, chromosomal amplifications in 17q12, 17q21.33-q25, 8p11.2, and 8q24.3 have poor to medium correlations with each other (Table S4), suggesting that the presence and effects of each amplicon may be functionally independent.

### Presence of Amplicons is Associated with Poor Outcome in ER+ Breast Cancers in Multiple Independent Datasets

**Figure 2:**
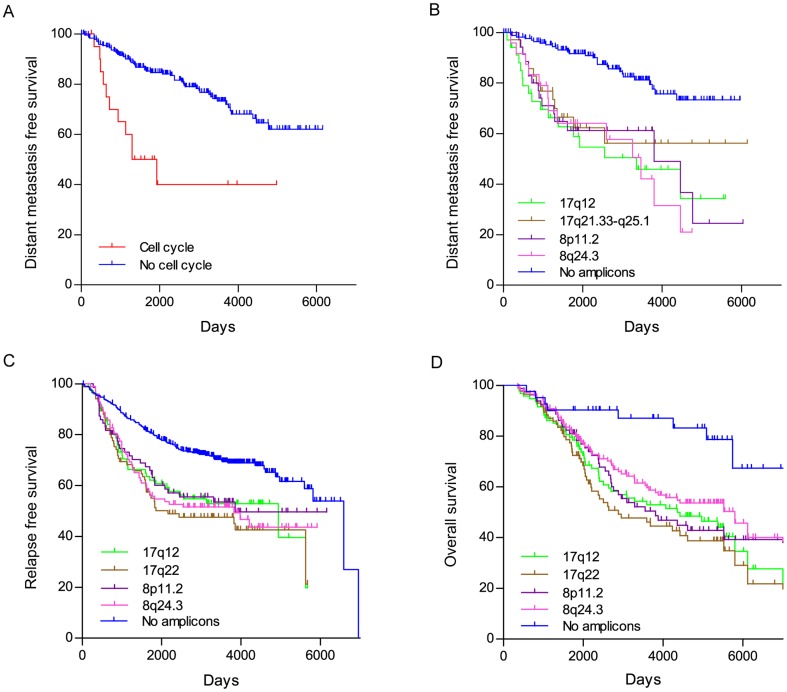
Patients with cell cycle pathway activation or outliers patterns consistent with amplification of 17q12, 17q21.33-q25.1, 8p11.2 and 8q24.3 show poor outcome under tamoxifen treatment. A) Kaplan-Meier curves of the samples in the primary dataset (GSE6532) enriched for over-expressed cell cycle genes versus the rest of samples that don’t show this feature. Patients with cell cycle activated genes show a significant decrease in distant metastasis free survival rate (HR  = 9.71, 95% CI  = 3.3–28.6; P<0.0001). B) Kaplan-Meier curves of the ER+ samples in the primary dataset (GSE6532) stratified by presence of putative amplicons in 17q12, 17q21.33-q25.1, 8p11.2 and 8q24.3. Patients that show any one of the chromosomal amplifications have significantly higher relapse rates when compared to samples without any amplifications: 17q12 (HR  = 4.09, 95% CI  = 3.84–21.99; P  = 6.3e−07), 17q21.33– q25.1 (HR  = 3.14, 95% CI  = 2.17–13.62; P  = 3.0e−04), 8p11.2 (HR  = 3.75, 95% CI  = 3.18–18.31; P  = 5.7e−06), and 8q24.3 (HR  = 4.29, 95% CI  = 4.32–34.08; P  = 2.2e−06). C) Analysis of combined gene expression data of 624 ER+ breast cancers from multiple published data sets. Outlier analysis was performed to identify cases with evidence of amplification at 17q12, 17q22, 8p11.2, and 8q24.3 and those without evidence of any amplification. Kaplan-Meier curves of relapse free survival for ER+ samples with each of the four amplicons, and samples containing no amplicon are plotted: 17q12 (HR  = 2.30, 95% CI  = 1.45–3.64; P  = 4.0e−04), 17q22 (HR  = 3.07, 95% CI  = 1.99–4.73; P<1.0e−04), 8p11.2 (HR  = 1.96, 95% CI  = 1.23–3.13; P  = 4.9e−3), 8q24.3 (HR  = 2.38, 95% CI  = 1.60–3.55; P<1.0e−04) D) Kaplan-Meier curves of overall survival for the ER+ samples in the test CGH dataset (GSE22133) with each of the 4 amplicons, as well as samples that don’t have any of the chromosomal amplifications. Analysis of the CGH data identified amplification peaks at each of the four regions that overlap with the previously identified loci. Patients that show any one of the chromosomal amplifications have significantly higher event rates than those without any of the amplifications: 17q12 (HR  = 2.61, 95% CI  = 1.51–5.51; P  = 6.8e−04), 17q22 (HR  = 3.02, 95% CI  = 1.76–5.18; P  = 7.3e−05), 8p11.2 (HR  = 2.65, 95% CI  = 1.48–4.74; P  = 1.3e−03), and 8q24.3 (HR  = 2.12, 95% CI  = 1.24–3.65; P  = 6.7e−03). Log-rank tests were used to calculate all the P values.

The effects of the presence of outliers in the cell cycle pathway, and of each of the four amplicons on distant metastasis free survival (DMFS) was determined. Presence of cell cycle pathway genes was found associated with significantly lower DMFS (log-rank P  = 0.0013), as well as higher hazard ratio (HR  = 9.71, 95% CI  = 3.3–28.6) in ER+ breast cancers, compared to tumors that lack this signature (Figure 2A). Presence of any of the four amplicons was also associated with lower DMFS compared to tumors without amplicons (Figure 2B). Hazard ratios for samples with amplicons on 17q12, 17q21.33-q25, 8p11.2 or 8q24.3 vs. no amplicons were 4.09, 3.14, 3.75, and 4.29 respectively, while log-rank P values for the DMFS differences were 6.3e−07, 3.0e−04, 5.7e−06, and 2.2e−06.

For validation, we first analyzed a data set of 624 early stage ER+ breast cancers for which relapse free survival data was available. This dataset included patients from over 20 published studies for whom gene expression data were combined as previously described [37]. Samples from the training set (GEO accession number GSE6532) were specifically excluded from this combined data. The clinical characteristics of the rest of the samples are listed in Table S5. This gene expression data was analyzed to identify breast cancers that had outlier patterns consistent with presence of the amplicons in 17q12, 17q21.33-q25, 8p11.2, and 8q24.3, as described in the Methods section. Kaplan-Meyer curves of the survival fraction for ER+ cases showed that samples which scored as having any of the putative amplicons, as assayed by outlier analysis, had significantly poorer relapse free survival compared to samples having no amplicons (Figure 2C), validating our results.

In these datasets, the amplicons were imputed by analysis of gene expression outliers in regions of known amplification. To test whether the presence of genomic amplification is directly responsible for these findings, a separate CGH array dataset [38] (GEO accession number GSE22133) with 359 samples and 8.1 years of median follow-up survival information was analyzed. We retained only the 222 ER+ samples for our analysis (Table S6). Although we expect that the ER+ cancers would have received adjuvant tamoxifen therapy, patients were not uniformly treated and specifics of the exact treatments were unavailable for this dataset. Copy number estimates obtained from GEO were segmented using circular binary segmentation (CBS) [39], followed by identification of significant amplification peaks with the GISTIC [40] algorithm as described in [38]. Amplification peaks were detected in 17q12, 17q22, 8p11.2 and 8q24.3 which overlapped the regions previously found by gene expression analysis. Correlation analysis between samples with these amplicons showed little to medium associations (Table S7) similar to the previously obtained values in Table S4.

Overall survival curves for samples with/without amplifications of 17q12, 17q22, 8p11.2 and 8q24.3 regions were determined using GISTIC and Kaplan-Meier estimator, and are shown in Figure 2D. This analysis showed that the presence of an amplicon in any of these four regions is associated with significantly worse outcome. Hazard ratios for samples with amplicons in 17q12, 17q22, 8p11.2 and 8q24.3 vs. no amplicons were 2.61, 3.02, 2.65, and 2.12 respectively, while log-rank P values for the survival difference were 6.8e−04, 7.3e−05, 1.3e−03, and 6.7e−03. Of note, 17q22 as identified by GISTIC, is a peak region included in the previously defined amplicon 17q21.33-q25 which contains a considerable number of outlier genes in the 17q22 locus (see Table 1).

### Associations between Presence of Amplicons and Oncotype Dx Recurrence Score

A validated marker of poor outcome in ER+ breast cancers with hormonal treatment is the Oncotype Dx assay [7]. This assay uses a weighted, linear combination of the expression of 21 genes to generate a single recurrence score RS. The genes used in this assay consist of HER2, GRB7, GSTM1, CD68, BAG1, invasion markers (MMP11, CTSL2), proliferation markers (Ki67, STK15, Survivin, CCNB1, MYBL2) as well as estrogen and reference markers. We used this gene panel and to generate a relative Oncotype Dx Recurrence Score using normalized expression levels and published weights [7]. This calculation of a relative Oncotype Dx recurrence score from gene expression array data is based on prior studies which have such relative scores to correlate with both outcome and molecular subtype [9], [41]. This relative RS score was able to separate poor prognosis samples from good prognosis samples in the tamoxifen treated sample set of 268 patients, validating this relative ODx score as being prognostic(Figure S2). We found (Figure S3) that the presence of any of these amplicons was associated with higher recurrence scores, while ER+ cancers lacking the amplicons had lower recurrence scores. A potentially significant finding was that the relative Oncotype Dx scores of tumors with amplicons 17q21.33-q25.1, 8p11.2 and 8q24.3 was lower than that of tumors with HER2 amplification (Figure S3), while their prognosis was similarly poor (Figure 2B). This observation suggests that Oncotype Dx may, in some cases, underestimate the risk of poor prognosis in tumors with these amplifications, and that some of tumors classified as “intermediate risk” by Oncotype Dx may in fact be high risk tumors.

To further test the hypothesis that regions 17q21.33-q25.1, 8p11.2 and 8q24.3 are likely to be amplified in ER+/HER2- breast cancer samples having high Oncotype Dx recurrence scores because of upregulation of cell-cycle genes, a set of 14 ER+/HER2- breast cancer samples with known Oncotype Dx scores was evaluated for the presence of 17q21.33-q25.1, 8p11.2 or 8q24.3 amplifications using FISH. Out of 14 samples, 8 had high recurrence scores (RS) (>30) and 6 had low scores (<18). As shown in Figure S4 and Table 2, cancers with high RS had amplification of at least one of these regions, while almost all cancers with low RS did not exhibit any amplification at these chromosomal locations.

**Table 2:** FISH scores for ER+/HER2- breast cancer tissue samples.

17q22	8q24.3	8p11.2	Oncotype Dx
amplified	amplified	amplified	46
not amplified	not amplified	amplified	42
borderline	not amplified	amplified	38
amplified	borderline	borderline	36
borderline	amplified	borderline	33
amplified	amplified	amplified	44
amplified	amplified	borderline	42
borderline	borderline	borderline	34
no signal	not amplified	not amplified	13
no signal	not amplified	not amplified	8
not amplified	not amplified	not amplified	5
borderline	not amplified	not amplified	12
not amplified	no signal	no signal	11
not amplified	not amplified	not amplified	11

Fluorescence in situ hybridization (FISH) results for 14 paraffin embedded ER+/HER2- breast cancer samples. Scores were calculated as the average number of spots over 20 cancer cells for each chromosomal location and separated into amplified, not amplified and borderline classes as follows: (>4 amplified; 2–4 borderline; <2 not amplified). The last column lists the associated Oncotype Dx score for each sample, 8 have high scores (>31) while 6 have low scores (<18).

### Associations between Presence of Amplicons and Histologic Grade

Histologic grade is also a strong predictor of outcome in ER+ breast cancer, with low grade tumors having good outcome with hormone therapy and high grade tumors having poor outcome in this setting [42]. In order to rule out the possibility that the presence of the amplicons is a surrogate for high histologic grade, a multivariate Cox analysis (Table 3) was performed on the training data set (GEO accession number GSE6532) to explore the relation between the presence of any of the four amplicons and other clinical markers (patient age, tumor size, node status, tumor grade and HER2 status) as well as the relative Oncotype Dx score calculated from gene expression data. We found that the presence of amplicons was a significant predictor of distant metastasis (HR  = 2.53, P  = 0.0067), more so than, tumor size (HR  = 1.38, P  = 0.0180), histologic grade (HR  = 0.44, P  = 0.0959) or ODx RS (HR  = 1.08, P  = 0.3838). If the amplicon covariate was removed from the Cox analysis, then significant predictors of distant metastasis become ODx RS (HR  = 1.19, P  = 0.0487) and tumor size (HR  = 1.29, P  = 0.0330).

**Table 3:** Multivariate Cox analysis of Age, Tumor size, Tumor grade, Lymph node status, Progesteron status, Oncotype Dx recurrence score, Her2 amplicon (17q12) and Any amplicon (17q12, 17q21.33-q25.1, 8p11.2 or 8q24.3).

Covariate	P values	Hazard ratio (95% CI)	
**Analysis without the combined amplicons**			
Age	0.8935		1.00 (0.97–1.03)
Tumor size	0.0330		1.29 (1.02–1.62)
Low grade	0.0688		0.41 (0.16–1.07)
High grade	0.2577		0.67 (0.33–1.34)
Lymph node negative	0.3866		0.77 (0.43–1.39)
Onctoype Dx recurrence score	0.0487		1.19 (1.00–1.41)
Her2 amplicon	0.9245		1.05 (0.39–2.82)
**Analysis with the combined amplicons**			
Age	0.9853		1.00 (0.97–1.03)
Tumor size	0.0180		1.38 (1.05–1.70)
Low grade	0.0959		0.44 (0.17–1.15)
High grade	0.2372		0.66 (0.34–1.31)
Lymph node negative	0.4073		0.78 (0.43–1.40)
Onctoype Dx recurrence score	0.3838		1.08 (0.91–1.29)
Her2 amplicon	0.8021		0.89 (0.35–2.23)
Any amplicon	0.0067		2.53 (1.30–4.93)

219 samples from the primary data set (GSE6532) had clinical information for all analyzed covariates. Cox proportional-hazard regression was performed on the reduced data set (with and without ‘Any amplicon’ covariate) resulting in a significant overall model fit (P  = 0.0005 and P  = 0.0001).

**Figure 3:**
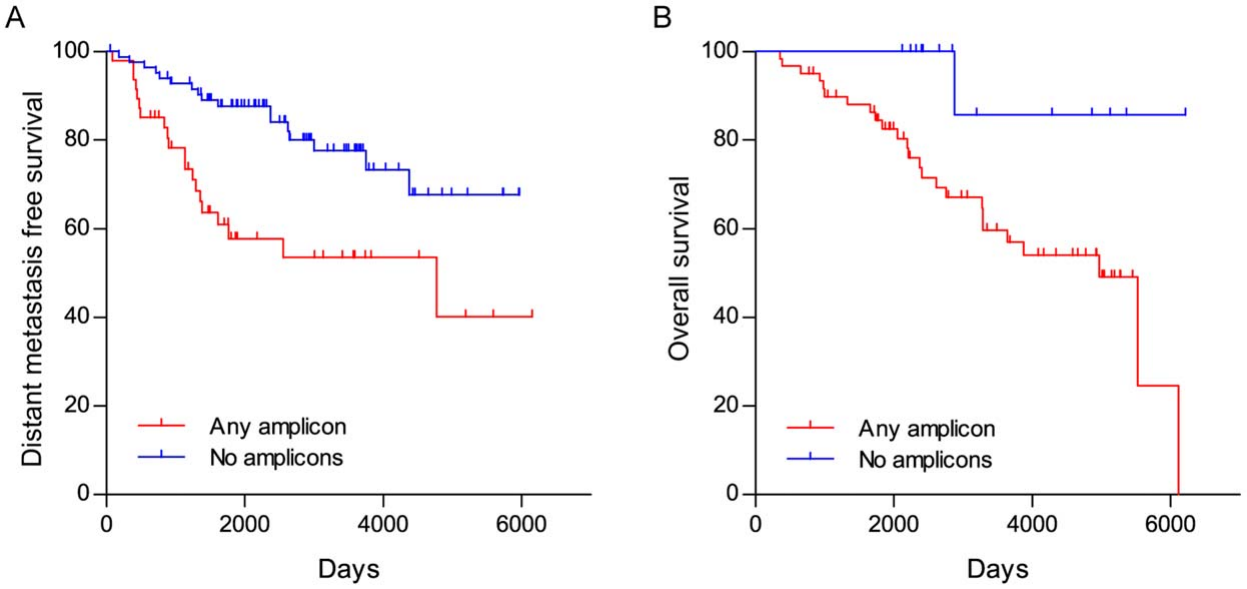
Analysis of intermediate grade tumors by presence of amplicons. Kaplan-Meier curves comparing distant relapse rates for intermediate grade cancers with any of the 4 amplicons versus cancers with none of the amplicons (A) in the training set GSE6532 (HR  = 3.22, 95% CI  = 1.6–6.5; P  = 0.0012). Also shown Kaplan-Meier curves comparing overall survival for intermediate grade cancers with any of the 4 amplicons versus cancers with none of the amplicons (B) in the test set GSE22133 (HR  = 3.01, 95% CI  = 1.2–7.6; P  = 0.0200).

We also analyzed the ability of the presence of any amplicon to discriminate outcome in intermediate grade tumors, which is a clinical grade category with unclear prognostic significance. Two datasets, (GSE6532 training set with gene expression data, and GSE22133 validation set with CGH data) where annotated pathologic grade information was available were analyzed. Kaplan-Meier curves comparing distant relapse rates for intermediate grade tumors with any of these four amplicons versus cases with none of the amplicons (Figure 3A) in the training set GSE6532, were significantly different (HR  = 3.22, 95% CI  = 1.6–6.5; P  = 0.0012). This was true also for Kaplan-Meier curves comparing overall survival for intermediate grade cancers with any of the 4 amplicons versus cases with none of the amplicons (Figure 3B) in the test set GSE22133 (HR  = 3.01, 95% CI  = 1.2–7.6; P  = 0.0200). Together, these results demonstrate that the amplicon associated risk categories have a discriminatory power beyond that of standard histologic grade.

### FISH-based Assay has Potential Prognostic Value in ER+/HER2- Breast Cancers Treated with Hormone Therapy

**Figure 4:**
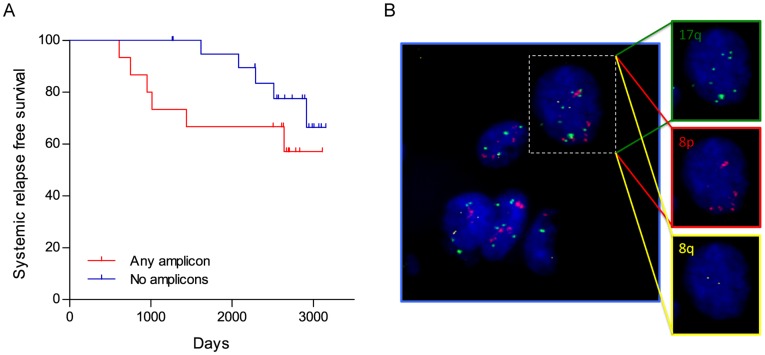
Analysis of amplicon status using multiplexed FISH in a cohort of tamoxifen treated ER+/HER2- breast cancers. ER+/HER2- samples with systemic relapse events were probed for the amplification of 8p11.2, 17q22 and 8q24.3 by multiplexed FISH assay as described in Methods. A) Out of 36 samples, 15 had at least one region amplified. Kaplan-Meier curves for relapse free survival is shown for cancers having at least one amplicon vs cancer having no amplicons (HR  = 2.31, 95% CI  = 0.66–8.06; P  = 0.1041 (Gehan-Willcoxon) or P  = 0.1886 (Mantel-Cox)). B) A typical image of multicolor FISH in a breast cancer specimen. This cell has evidence of amplification of both 17q23.1 and 8p11 loci, but normal 8q24 loci.

A multiplexed FISH assay to detect 8p11.2, 17q22 and 8q24.3 amplicons in FFPE sections was developed using prelabeled FISH probes from validated BACs (Bacterial Artificial Chromosomes). The specificity of each probe was tested on metaphase chromosome spreads and hybridized to the corresponding chromosomal locations. The FISH assay was applied to 36 ER+/HER2− samples from the MicMa cohort that were treated with adjuvant hormonal therapy [20] (Figure 4). Tumor samples, present in tissue microarray format, were scored for amplification of each amplicon by averaging signals in 20 tumor cells/sample. The KM curves for systemic relapse free survival in this cohort for patients with and without any amplicon are shown in Figure 4. There is a trend for decreased relapse-free survival in patients scored as having any amplicon, vs. having no amplicon, but given the small sample size, this did not achieve statistical significance (P = 0.1041). The thresholds for amplification were optimized using the outcomes in this sample set, and thus require independent validation in future studies. Of note, very few relapses occurred earlier than 1500 days in the no-amplicon group, whereas the majority of relapse in the any-amplicon group occurred before 1500 days.

## Discussion

Currently Oncotype Dx assays are routinely used to predict outcome and guide treatment for early stage ER+/HER2- breast cancer patients in the US. A high Oncotype Dx recurrence score can identify patients likely to have poor outcome with hormonal therapy alone, and who may benefit most from the addition of chemotherapy. However, such prognostic assays are expensive, often have intermediate risk scores with unclear predictive value, do not give biological insight into mechanisms driving poor prognosis, and do not identify potential therapeutic targets.

The results presented in this paper demonstrate that the presence of amplifications in chromosomal regions 17q21.33-q25.1, 8p11.2 and 8q24.3 are strong markers of poor prognosis in ER+/HER2- breast cancers. Our results suggest that these amplicons may function in a manner similar to HER2 amplification in identifying ER+ breast cancers with poor outcome and relative resistance to hormone therapy. In our primary dataset of 268 patients treated with tamoxifen, of the 44 patients who suffered distant metastasis within the first 4 years after diagnosis, only 30% were identified as having only 17q12 (HER2+) amplification, while an additional 42% had amplification of one of the three other amplicons. These results suggest that the presence of other amplicons, besides HER2, is associated with early relapse in ER+ breast cancer.

The presence of these amplicons is also associated with higher expression of proliferative genes/cell cycle genes that drive a high Oncotype Dx (ODx) recurrence score. Direct analysis of clinical specimens for amplification of these regions using FISH also demonstrated that the presence of amplification in each locus is associated with high ODx scores, while tumors that lack any of the amplicons have low recurrence ODx scores. This indicates that identification of chromosomal amplifications in defined regions, by a cost effective FISH assay, may be a clinically useful biomarker for predicting poor outcome in early stage ER+/HER2- breast cancers. Moreover, analysis of relative ODx scores in gene expression data sets suggests that ODx may underestimate risk associated with presence of amplicons, and that some tumors classified as intermediate risk by ODx assay may instead be poor prognosis tumors marked by a high risk amplicon. These finding will need to be validated in future studies analyzing a larger set of ER+ breast cancers with known ODx scores and clinical outcome.

In addition to their usefulness as biomarkers of risk in ER+ breast cancers, the chromosomal regions 17q21.33-q25.1, 8p11.2 and 8q24.3 contain oncogenes that may be valuable as therapeutic targets for novel drug therapies. Genes in the 8p11.2 region identified as putative oncogenes and therapeutic targets include U6 snRNA-associated Sm-like protein (LSM1), Wolf-Hirschhorn syndrome candidate 1-like 1 (WHSC1L1), and the RNA Polymerase III subunit BRF2 in region 8p11.2. Of note BRF2 has the highest associated HR, and was recently identified as a putative oncogene in squamous cell lung cancer [29], [30], [34], [43]. Fibroblast growth factor receptor-1 (FGFR1), recently identified as a putative driver of endocrine resistance in breast cancer [36], is at the edges of the 8p11.2 amplicon that we found associated with early relapse in ER+ breast cancer treated with tamoxifen. Its outlier profile is associated with poor survival with hazard ratio of 1.8 and a log-rank P value of 0.046 (Table S2). However, in our analysis FGFR1 is not the strongest candidate in this region, and was not present in our minimal amplicon region defined by our methods.

As seen in Figure S1, the majority of outlier genes associated with poor prognosis on the *q* arm of chromosome 8 are clustered in the region 8q24.3 with the rest of them scattered all the way to 8q11.2. This suggests that in some cases the whole *q* arm of chromosome 8 is amplified or that there are a number of amplicons on 8q that correlate with 8q24.3. Slightly more upstream of 8q24.3 there is a well known oncogene MYC, a key estrogen effector, that has been reported to induce tamoxifen resistance when over-expressed [44]. Although MYC could also contribute to the effect of this amplicon on early relapse, it was not identified in our analysis as strongly associated with differential survival (log-rank P  = 0.042, Table S2) compared to more distal genes, suggesting it may contribute to only a minority of cases containing this amplicon. Other potential genes driving tumorigenesis in 8q24.3 include Heat Shock Transcription Factor 1 (HSF1), which has been shown to be a powerful potentiator of tumorigenesis [29], [30], [34]. Also of interest is YWHAZ, a member of the 14-3-3 family of proteins. High expression of YWHAZ has been associated with poor clinical outcome in ER+ breast cancer [45]. Down-regulation of YWHAX can restore tamoxifen sensitivity to tamoxifen resistant populations of MCF-7 cells, suggesting it may play a direct role in mediating hormone resistance [46].

Of the chromosomal regions identified in this study, 17q21.33-q25.1 is the least understood. Situated downstream of a much better known amplicon 17q12 (HER2+), it is known to be amplified and correlated with high grade tumors and poor prognosis [26]. However, there is still no definite identification of driver oncogenes in this region. Possible candidates are CLTC, involved in gene fusions in B-cell lymphomas and non-small cell lung carcinomas, and RAD51C involved in DNA repair and homologous recombination. The gene with highest HR for relapse in this region is Prohibitin (PHB), a transcriptional regulator that has been shown to have both oncogenic and tumor suppressor capability in different contexts. Of note PHB has been reported to associate with and inhibit ER-mediated transcriptional function, suggesting it may play a role in modulating ER-function [47].

Another gene associated with early relapse is Cyclin D1 (CCND1, log-rank P  = 5.7e-06, Table S2) [48] located on chromosomal band 11q13, which is another well known amplification site [49]. However CCND1 is also a cell cycle marker and its expression is associated with proliferation. Thus the association of high CCND1 expression with poor outcome may in part reflect its role in proliferation and not just as a driver oncogene. This region was not identified as an independent potential amplicon in our analysis. Intriguingly there are reports of an association between 11q13 amplification and amplification of 8p12 [38], [50], [51] in breast cancers, with some reports demonstrating a physical association between these domains [50].

Analysis for the presence of 17q21.33-q25.1, 8p11.2 and 8q24.3 amplicons by a multiplexed FISH assay in 36 ER+/HER2- samples from the MicMa cohort showed there is a trend towards decreased relapse free survival in patients who have amplicons compared to those who do not. Although small numbers limit statistical significance, these results are encouraging and suggest that a FISH-based assay could be developed as a prognostic tool. Future studies that evaluate large, well annotated clinical data sets are necessary to validate the FISH assay and determine whether these amplicons can be of used as predictive and prognostic markers in ER+ breast cancer.

In summary, the data presented here suggest that amplification of chromosomal regions 17q21.33-q25.1, 8p11.2 and 8q24.3 is strongly associated with early relapse in early stage ER+/HER2- breast cancers treated with hormonal therapy, and correlates with high Oncotype Dx recurrence scores. These chromosomal regions also contain genes whose over-expression may directly drive early relapse and/or hormone independence in ER+ breast cancers, and may be candidates for targeted therapy. Assays to identify the presence of amplicons may then both identify patients at high risk of relapse with hormonal therapy alone, and also potentially help determine what targeted therapy may be most appropriate to improve outcome in these poor prognosis cancers.

## Materials and Methods

### Ethics Statement

Clinical samples obtained at CINJ-UMDNJ were pre-existing archived samples that were de-identified and obtained without individual consent under a protocol approved by the Institutional Review Board of UMDNJ (Piscataway/NewBrunswick Campus). Samples from Radium Hospital for which clinical outcome data were available were obtained with written patient consent under a protocol approved by Regional Ethical Committee of South Eastern Norway (REK sør-øst).

### Data Processing

Three breast cancer gene expression datasets from Loi et al. [12], [21] were downloaded from (GEO:www.ncbi.nlm.nih.gov/geo, accession number GSE6532). The sets are abbreviated as KIT, OXFT and GUYT representing the institutions of origin: Uppsala University Hospital, John Radcliffe Hospital, and Guys Hospital. They comprised of data from 81, 109 and 87 ER+ breast cancer samples from patients treated with tamoxifen with 9 years median clinical follow-up on Affymetrix U133A/B (KIT & OXFT) and U133Plus2 (GUYT) platforms. After MAS5 normalization, probes were retained only for genes found on both platforms. Expression values were log2 transformed and multiple probes/gene compressed to the probe with highest median expression across samples.

### Supervised Outlier Analysis of Gene Expression Datasets

Expression values were median centered and divided by the median absolute deviation (MAD) as described in Tomlins et al. [52]. This step was performed separately for KIT, OXFT and GUYT datasets to avoid distribution biases. Outlier low/high cut-off values for each gene were defined as those which were outside the 10/90% quantile cutoffs across samples (results were insensitive to varying the quantile cut-off by +/−5%). High/low outlier genes for each sample array were identified using these cutoffs. The dataset is now reduced to three binary matrices of size N_genes_ x N_samples_, one matrix for non-outliers and one each for high and low outliers. This process was implemented separately for each dataset (KIT, OXFT, GUYT) and the resulting matrices merged by concatenation over samples.

The high/low outlier matrices *B*_1_ and *B*_2_, with entries 1/0 if *gene i in sample j* was/was-not an outlier, were analysed further. Genes with <10 outliers across samples were discarded as not informative for statistical inference. For each remaining gene, the distribution of outliers across samples defines two classes: the sample set with “aberrant” (outlier) expression and the sample set with “normal” expression. Kaplan-Meier curves were used to identify the genes where these classes had a significant differential survival based on a log-rank test at p<0.05 (complete list in Table S2).

### Identification of Predictive Gene Patterns for Tamoxifen Sensitivity

Do the outlier genes defined as above represent gene categories of clinical interest? For this to be true and statistically significant, sets of genes must exist with similar outlier classes - i.e., they must be over/under-expressed in roughly the same set of samples. This corresponds to identifying tightly correlated clusters of outlier genes and samples in the binary matrices *B*_1_ or *B*_2_. These were identified using the Phi coefficient (equivalent to a Pearson correlation between rows of matrices *B*_1_ or *B*_2_) as follows:

Let *C*_1_ and *C*_2_ be the covariance matrices between the rows of *B*_1_ and *B*_2_ respectively. Then, 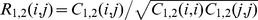 is the matrix of correlation coefficients between the outlier profiles of the genes in *B*_1_ or *B*_2_. Clusters of tightly correlated genes were identified by iteratively removing *row i* and *column i* with 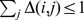 where 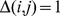 if 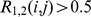 and 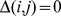 otherwise, until a stable set was obtained. Here, stability means that the size of the reduced matrix *R*’ stops changing. PCA plots of the resulting reduced matrices *B_1_* and *B_2_* were used to identify distinct groups of highly correlated genes for further analysis (eg. pathway enrichment [22]).

The identified genes were mapped to chromosomal locations and amplified regions identified using a sliding window 25 genes wide with a pace of 5 genes (varying the window size and/or pace by 5–10 genes did not affect results. The Fisher Exact test [53] was used to assess significance. The Benjamini-Hochberg method [54] was used to implement FDR <5% by converting p-values to q-values. For each array, chromosomal regions with q <0.05 were marked as potential amplifications and ordered by frequency in the cluster sample set.

### Relative Oncotype Dx Scores

The Relative Oncotype Dx score is calculated using normalized gene expression values of the set of genes from the original score together with their published weights [7]. The genes used in calculating this score are: HER2, GRB7, GSTM1, CD68, BAG1, invasion markers MMP11, CTSL2, proliferation markers Ki67, STK15, Survivin, CCNB1, MYBL2 and hormonal markers ER, PGR, BCL2, and SCUBE2. A separate score is calculated for each group and then combined in a final score:
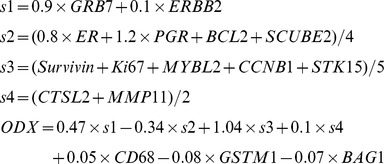



### Fluorescence in situ Hybridization (FISH)

Prelabeled FISH probes for BAC clones RP11-1065N2, RP11-90P5 and RP11-1136N16 were purchased (Empire Genomics, Buffalo, NY) and tested on metaphase chromosome spreads. FISH experiments were performed on 14 4 µm paraffin embedded breast cancer tissue slides, collected from ER+/HER2- breast cancer patients treated in 2007–2009 at Robert Wood Johnson University Hospital, New Jersey, USA. Hybridization was performed on hybrite for 16–24 hours at 37°C, and slides washed, first with 4x SSC for 3 min at 37°C then with 0.1% NP-40 (Vysis, Downers Grove, IL, USA) for 30 sec at room temperature. Slides were scored for chromosomal amplification by counting signals in 20 tumor cells and averaging.

A multiplex FISH assay was also developed to score the amplicons on specimens from 36 ER+/HER2- patients treated with hormone therapy from the MicMa cohort that were available in a tissue microarray format [55]. The probes were labelled by Nick translation with nucleotides labelled with Green-dUTP, Alexa Fluor 594-5-dUTP and HyPer5 dCTP, respectively. Scoring of FISH signals was done by acquiring z-stacks of the whole thickness of each sample and using a Nikon Ti microscope attached to a Yokogawa spinning-disk confocal unit. Non-tumor cells such as fibroblasts or lymphocytes were used as internal controls. The amplicon count for the sample was the average number of counts over 20 cells. We consider the sample to have an amplicon if its amplicon count is ≥3.5 for 8p11.2, ≥4.0 for 17q12 and ≥2.8 for 8q24.3. These thresholds were obtained by finding the optimal thresholds associated with survival difference between the cases that had at least one amplicon against the cases that had none in this sample set.

## Supporting Information

Figure S1 **Clustergram of the correlation matrix between selected over-expressed genes identify pathways and amplicons for poor survival under tamoxifen treatment.** The Phi coefficients between gene pairs of highly expressed outlier genes associated with tamoxifen resistance in Figure 1A produce a correlation matrix. The figure shows the resulting heatmap of this correlation matrix using hierarchical clustering using Pearson correlation distance and complete linkage. Genes in the same pathway or chromosomal region are clustered together as marked.
(TIF)

Figure S2 **Survival curves for samples with high/low Oncotype Dx scores.** Kaplan-Meier curves showing significantly lower survival (HR  = 2.81, 95% CI  = 1.7–4.5; P<0.0001) for tumor samples with high Oncotype Dx scores (ODx score >0) versus low Oncotype Dx scores (ODx score <0).
(TIF)

Figure S3 **Oncotype Dx and presence of amplicons in ER+ breast cancer.** Relative Oncotype Dx scores calculated across all 3 datasets (GSE6532) as outlined in Methods, are shown as mean values with standard errors for each group of samples listed on the vertical axes. Note that the Oncotype Dx scores for patients with the three novel amplicons are less than that for HER2 amplicon, in spite of their similar poor survival. This suggests that Oncotype Dx does not adequately assess the presence of these novel amplicons, and may underestimate risk in some cases.
(TIF)

Figure S4 **Analysis of amplicon status using FISH in a cohort of tamoxifen treated ER+/HER2− breast cancers.** This figure shows typical FISH images from analysis of FFPE slides for samples with/without amplicons using probes specific to each amplicon.
(TIF)

Table S1 **Clinical information file.** Excel 2003 file containing clinical characteristics of the breast tumor samples used in the gene expression analysis from the study by Loi et al. [12], [21]. Relative Oncotype Dx scores together with pathway/amplicon presence for each sample are also listed.
(XLS)

Table S2 **Survival associated with outlier genes.** Excel 2003 file containing a table of outlier association results for all genes used in the analysis. Along with the outlier scores and hazard ratios, corresponding P values are also listed.
(XLS)

Table S3 **Gene patterns associated with tamoxifen response.** Gene Ontology pathway/chromosomal location enrichment results in the primary gene expression dataset GSE6532. Significance was assessed using Fisher Exact Test.
(DOC)

Table S4 **Sample correlations between gene patterns associated with tamoxifen resistance.** Sample correlations between cell cycle pathway and amplicons associated with tamoxifen resistance in the primary gene expression dataset GSE6532. Values represent Phi coefficients measuring the strength of association between the group of samples that over-express cell cycle genes and amplicons 17q12, 17q21.33-q25.1, 8p11.2 and 8q24.3. The last column lists the percentage counts of ER+ samples with the associated pathway/amplicons. Highlighted in bold are correlation values significant at P<0.01 except for self correlations.
(DOC)

Table S5 **Clinical information file.** Excel 2003 file containing clinical characteristics of the breast tumor samples used in the analysis of the combined gene expression data set from the study by Györffy et al. [37]. Amplicon presence as found by the unsupervised outlier analysis are also listed.
(XLS)

Table S6 **Clinical information file.** Excel 2003 file containing clinical characteristics of the breast tumor samples used in the CGH data analysis. Amplicon presence as found by GISTIC is also listed for each sample.
(XLS)

Table S7 **Sample correlations between amplicons 17q12, 17q22, 8p11.2 and 8q24.3 in an independent CGH array data set.** Phi coefficients measuring the strength of association between amplicons 17q12, 17q22, 8p11.2 and 8q24.3 in the test CGH dataset GSE22133. The last column lists the percentage counts of ER+ samples with the associated amplicons. Highlighted in bold are correlation values significant at P<0.01 except for self correlations.
(DOC)
