# Supplementary figures and images for: Amplified Loci on Chromosomes 8 and 17 Predict Early Relapse in ER-Positive Breast Cancers
Source: PLoS One. 2012 Jun 13;7(6):e38575. doi: 10.1371/journal.pone.0038575 (PMC3374812; doi:10.1371/journal.pone.0038575)

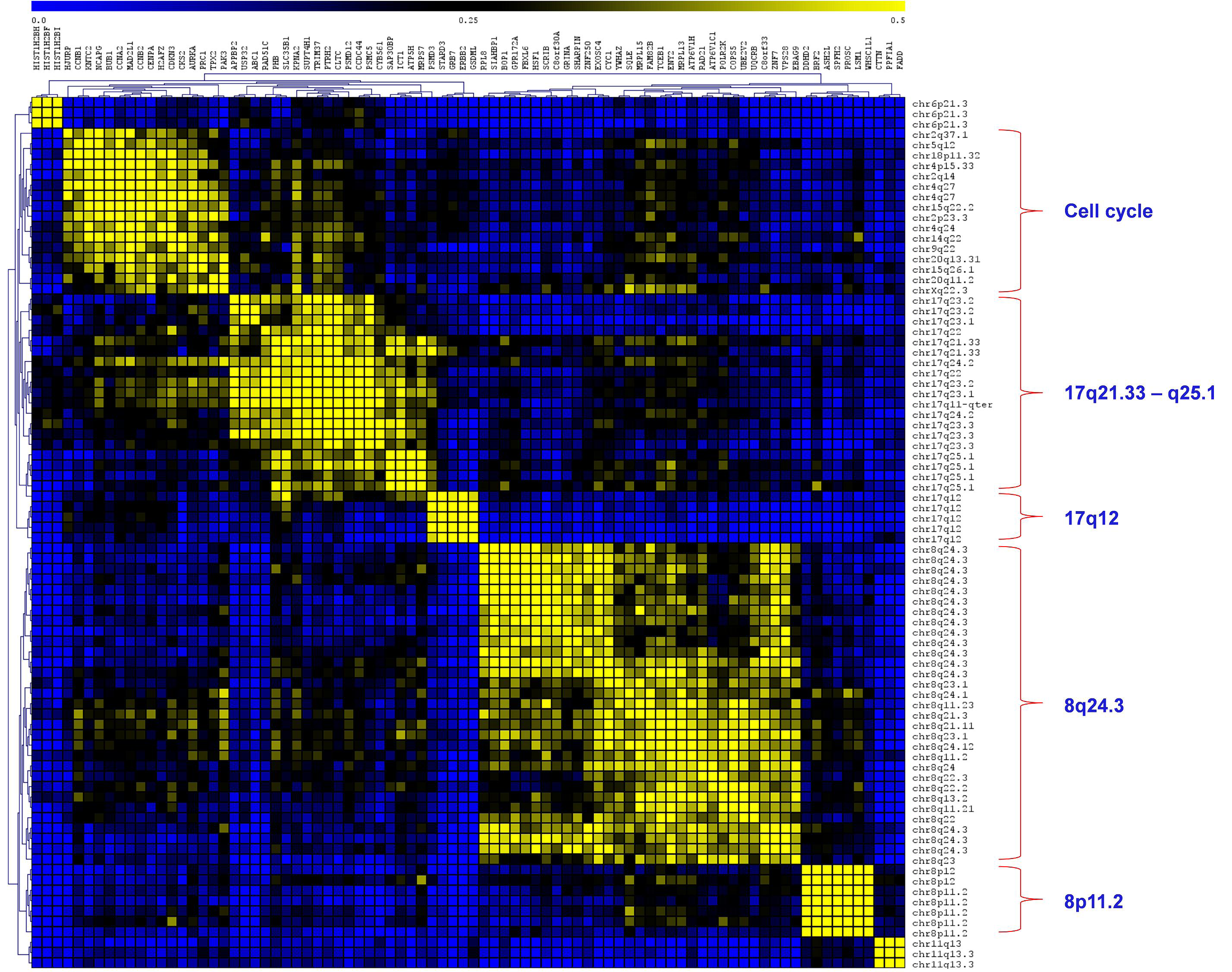

Supplement: Figure S1 — Clustergram of the correlation matrix between selected over-expressed genes identify pathways and amplicons for poor survival under tamoxifen treatment. The Phi coefficients between gene pairs of highly expressed outlier genes associated with tamoxifen resistance in Figure 1A produce a correlation matrix. The figure shows the resulting heatmap of this correlation matrix using hierarchical clustering using Pearson correlation distance and complete linkage. Genes in the same pathway or chromosomal region are clustered together as marked. (TIF) [file pone.0038575.s001.tif]

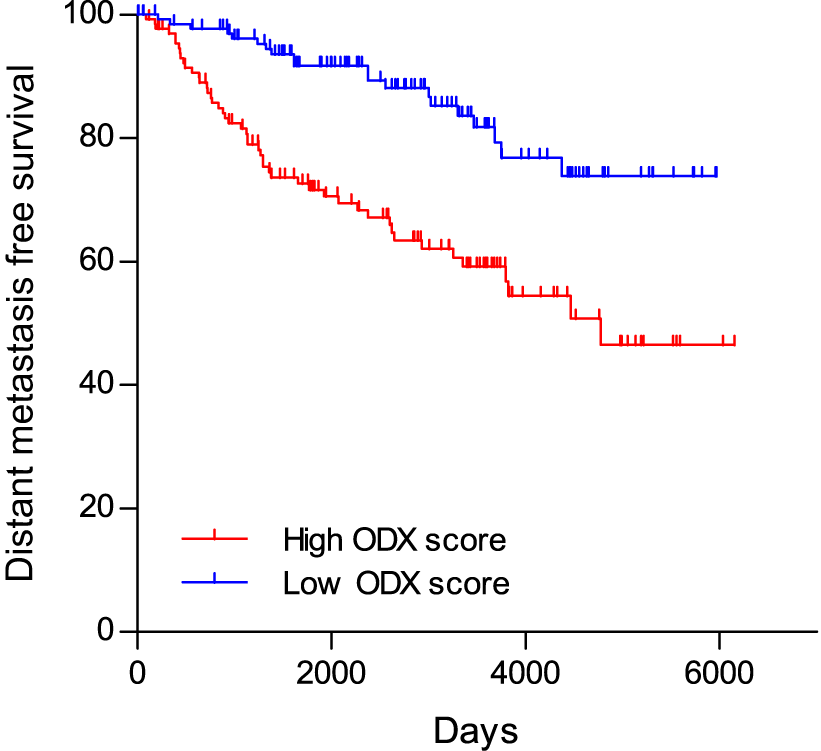

Supplement: Figure S2 — Survival curves for samples with high/low Oncotype Dx scores. Kaplan-Meier curves showing significantly lower survival (HR = 2.81, 95% CI = 1.7–4.5; P<0.0001) for tumor samples with high Oncotype Dx scores (ODx score >0) versus low Oncotype Dx scores (ODx score <0). (TIF) [file pone.0038575.s002.tif]

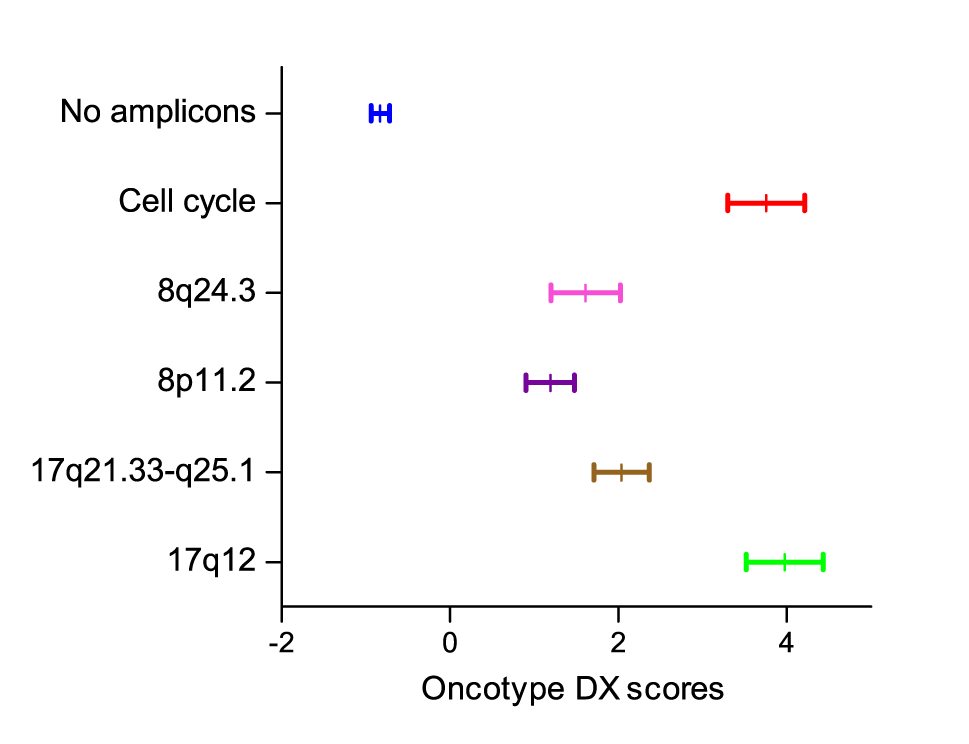

Supplement: Figure S3 — Oncotype Dx and presence of amplicons in ER+ breast cancer. Relative Oncotype Dx scores calculated across all 3 datasets (GSE6532) as outlined in Methods, are shown as mean values with standard errors for each group of samples listed on the vertical axes. Note that the Oncotype Dx scores for patients with the three novel amplicons are less than that for HER2 amplicon, in spite of their similar poor survival. This suggests that Oncotype Dx does not adequately assess the presence of these novel amplicons, and may underestimate risk in some cases. (TIF) [file pone.0038575.s003.tif]

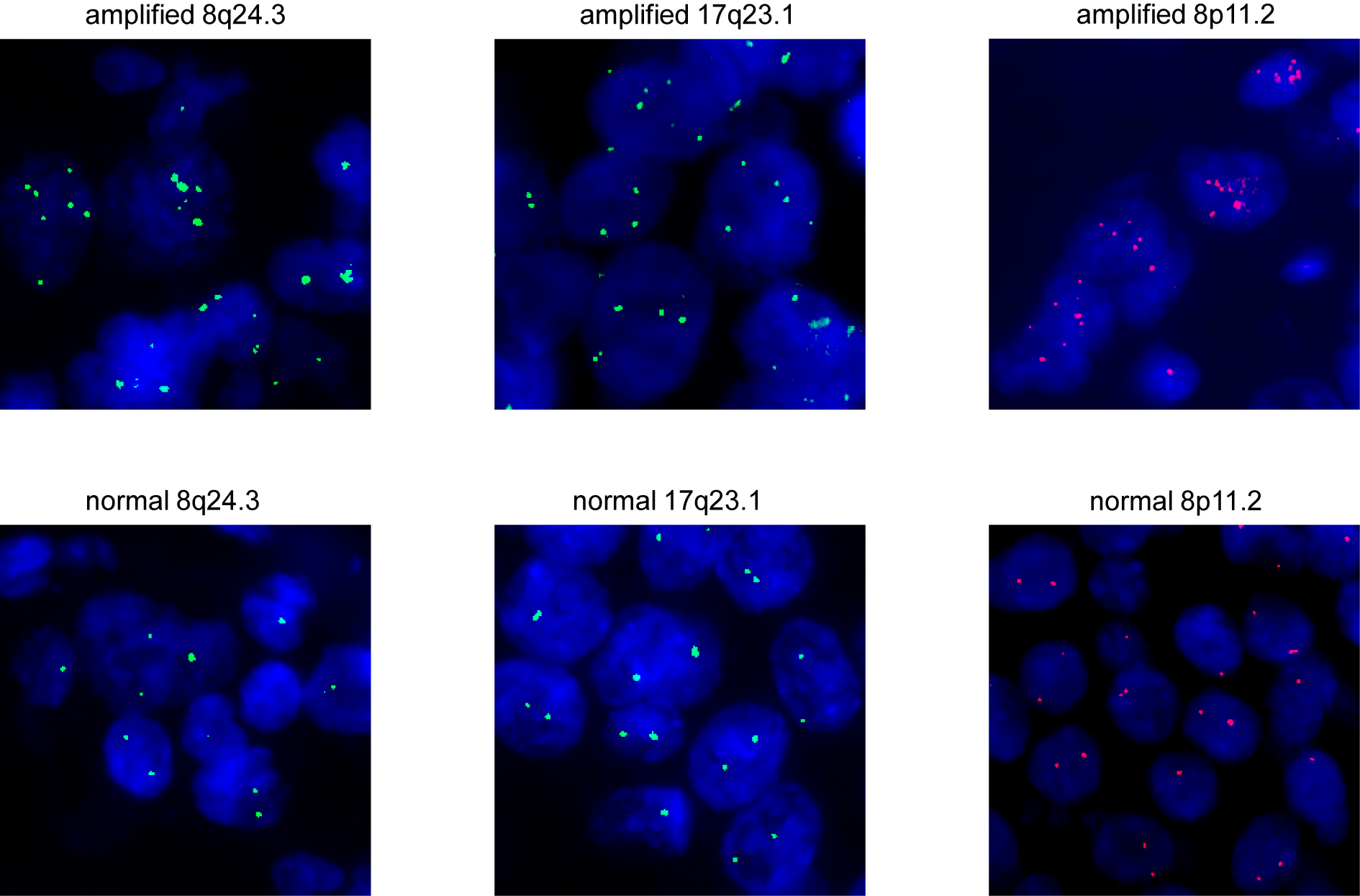

Supplement: Figure S4 — Analysis of amplicon status using FISH in a cohort of tamoxifen treated ER+/HER2− breast cancers. This figure shows typical FISH images from analysis of FFPE slides for samples with/without amplicons using probes specific to each amplicon. (TIF) [file pone.0038575.s004.tif]
