# Supplementary material for: Amplified Loci on Chromosomes 8 and 17 Predict Early Relapse in ER-Positive Breast Cancers
Source: PLoS One. 2012 Jun 13;7(6):e38575. doi: 10.1371/journal.pone.0038575 (PMC3374812; doi:10.1371/journal.pone.0038575)
Supplement: Table S3 — Gene patterns associated with tamoxifen response. Gene Ontology pathway/chromosomal location enrichment results in the primary gene expression dataset GSE6532. Significance was assessed using Fisher Exact Test. (DOC) [file pone.0038575.s007.doc]

**Table S3. Gene patterns associated with tamoxifen response**

Gene Ontology pathway/chromosomal location enrichment results in the primary gene expression dataset GSE6532. Significance was assessed using Fisher Exact Test.

|  | **Over-expression** | **P value** | **Under-expression** | **P value** |
| --- | --- | --- | --- | --- |
| **Good outcome** | Immune response | 1.61E-05 | Cell cycle | 1.10E-03 |
|  | Development | 7.56E-08 |  |  |
|  | Cell adhesion | 1.68E-04 |  |  |
| **Poor outcome** | Cell cycle | 9.12E-07 | Immune response | 1.36E-05 |
|  | 17q21.33-q25.1 | 3.87E-05 | Cell adhesion | 2.01E-08 |
|  | 17q12 | 1.39E-08 |  |  |
|  | 8p11.2 | 1.11E-16 |  |  |
|  | 8q24.3 | 2.22E-16 |  |  |
