# Supplementary material for: Amplified Loci on Chromosomes 8 and 17 Predict Early Relapse in ER-Positive Breast Cancers
Source: PLoS One. 2012 Jun 13;7(6):e38575. doi: 10.1371/journal.pone.0038575 (PMC3374812; doi:10.1371/journal.pone.0038575)
Supplement: Table S4 — Sample correlations between gene patterns associated with tamoxifen resistance. Sample correlations between cell cycle pathway and amplicons associated with tamoxifen resistance in the primary gene expression dataset GSE6532. Values represent Phi coefficients measuring the strength of association between the group of samples that over-express cell cycle genes and amplicons 17q12, 17q21.33-q25.1, 8p11.2 and 8q24.3. The last column lists the percentage counts of ER+ samples with the associated pathway/amplicons. Highlighted in bold are correlation values significant at P<0.01 except for self correlations. (DOC) [file pone.0038575.s008.doc]

**Table S4. Sample correlations between gene patterns associated with tamoxifen resistance**

Sample correlations between cell cycle pathway and amplicons associated with tamoxifen resistance in the primary gene expression dataset GSE6532. Values represent Phi coefficients measuring the strength of association between the group of samples that over-express cell cycle genes and amplicons 17q12, 17q21.33-q25.1, 8p11.2 and 8q24.3. The last column lists the percentage counts of ER+ samples with the associated pathway/amplicons. Highlighted in bold are correlation values significant at P < 0.01 except for self correlations.

|  | **Cell cycle** | **17q12** | **17q21.33-q25.1** | **8p11.2** | **8q24.3** | **Percent samples** |
| --- | --- | --- | --- | --- | --- | --- |
| **Cell cycle** | 1.00 | **0.26** | **0.30** | **0.17** | **0.20** | 7.8% |
| **17q12** | **0.26** | 1.00 | **0.18** | 0.01 | 0.00 | 12.7% |
| **17q21.33-q25.1** | **0.30** | **0.18** | 1.00 | 0.07 | **0.23** | 13.1% |
| **8p11.2** | **0.17** | 0.01 | 0.07 | 1.00 | **0.26** | 13.4% |
| **8q24.3** | **0.20** | 0.00 | **0.23** | **0.26** | 1.00 | 9.0% |
