# Supplementary material for: Amplified Loci on Chromosomes 8 and 17 Predict Early Relapse in ER-Positive Breast Cancers
Source: PLoS One. 2012 Jun 13;7(6):e38575. doi: 10.1371/journal.pone.0038575 (PMC3374812; doi:10.1371/journal.pone.0038575)
Supplement: Table S7 — Sample correlations between amplicons 17q12, 17q22, 8p11.2 and 8q24.3 in an independent CGH array data set. Phi coefficients measuring the strength of association between amplicons 17q12, 17q22, 8p11.2 and 8q24.3 in the test CGH dataset GSE22133. The last column lists the percentage counts of ER+ samples with the associated amplicons. Highlighted in bold are correlation values significant at P<0.01 except for self correlations. (DOC) [file pone.0038575.s011.doc]

**Table S7. Sample correlations between amplicons 17q12, 17q22, 8p11.2 and 8q24.3 in an independent CGH array data set**

Phi coefficients measuring the strength of association between amplicons 17q12, 17q22, 8p11.2 and 8q24.3 in the test CGH dataset GSE22133. The last column lists the percentage counts of ER+ samples with the associated amplicons. Highlighted in bold are correlation values significant at P < 0.01 except for self correlations.

|  | **17q12** | **17q22** | **8p11.2** | **8q24.3** | **Percent samples** |
| --- | --- | --- | --- | --- | --- |
| **17q12** | 1.00 | **0.32** | 0.12 | **0.27** | 51.8% |
| **17q22** | **0.32** | 1.00 | 0.15 | 0.20 | 41.9% |
| **8p11.2** | 0.01 | 0.15 | 1.00 | **0.22** | 45.9% |
| **8q24.3** | **0.27** | 0.20 | **0.22** | 1.00 | 68.5% |
